# The vacuolar anti-*Pseudomonal* activity of neutrophil primary granule peptidyl-arginine deiminase enzymes

**DOI:** 10.3389/fimmu.2024.1452393

**Published:** 2024-10-18

**Authors:** Rory Baird, Azeez Yusuf, Luke Forde, Kerstin Pohl, Kevin Kavanagh, Fidelma Fitzpatrick, Debananda Gogoi, Emer P. Reeves

**Affiliations:** ^1^ Pulmonary Clinical Science, Department of Anaesthesia and Critical Care Medicine, Royal College of Surgeons in Ireland, Beaumont Hospital, Dublin, Ireland; ^2^ Department of Biology, Maynooth University, Maynooth, Kildare, Ireland; ^3^ Department of Clinical Microbiology, Royal College of Surgeons in Ireland, Dublin, Ireland; ^4^ Department of Microbiology, Beaumont Hospital, Dublin, Ireland

**Keywords:** neutrophils, phagocytic vacuole, primary granules, peptidyl-arginine deiminases, bactericidal permeability increasing protein, antimicrobial activity

## Abstract

The role of neutrophils in host defense involves several cell processes including phagocytosis, degranulation of antimicrobial proteins, and the release of neutrophil extracellular traps (NETs). In turn, dysregulated cell activity is associated with the pathogenesis of airway and rheumatic diseases, in which neutrophil-derived enzymes including peptidyl-arginine deiminases (PADs) play a role. Known physiological functions of PADs in neutrophils are limited to the activity of PAD isotype 4 in histone citrullination in NET formation. The aim of this study was to extend our knowledge on the role of PADs in neutrophils and, specifically, bacterial killing within the confines of the phagocytic vacuole. Human neutrophils were fractionated by sucrose gradient ultracentrifuge and PADs localized in subcellular compartments by Western blot analysis. Direct interaction of PADs with *Pseudomonas aeruginosa* (*P. aeruginosa*) was assessed by flow cytometry and Western blot overlay. The participation of neutrophil PAD2 and PAD4 in killing of *P. aeruginosa* was assessed by inclusion of PAD-specific inhibitors. *In vitro*, bactericidal activity of recombinant human PAD2 or PAD4 enzymes against *P. aeruginosa* was determined by enumeration of colony-forming units (CFU). Together with neutrophil elastase (NE), PAD2 and PAD4 were localized to primary granules and, following activation with particulate stimuli, were degranulated in to the phagocytic vacuole. *In vitro*, PAD2 and PAD4 bound *P. aeruginosa* (p = 0.04) and significantly reduced bacterial survival to 49.1 ± 17.0 (p < 0.0001) and 48.5 ± 13.9% (p < 0.0001), respectively. Higher antibacterial activity was observed at neutral pH levels with the maximum toxicity at pH 6.5 and pH 7.5, comparable to the effects of neutrophil bactericidal permeability increasing protein. In phagosomal killing assays, inclusion of the PAD2 inhibitor, AFM-30a, or PAD4 inhibitor, GSK484, significantly increased survival of *P. aeruginosa* (AFM-30a, p = 0.05; and GSK484, p = 0.0079). Results indicate that PAD2 and PAD4 possess antimicrobial activity and are directly involved in the neutrophil antimicrobial processes. This study supports further research into the development of PAD-based antimicrobials.

## 1 Introduction

Neutrophils are one of the first immune cells to be recruited to the site of both bacterial and fungal infection. To advance our understanding of the immunity afforded by this circulating cell, studies have focused on the molecular basis of rare congenital disorders, including defects in neutrophil respiratory burst activity in patients with chronic granulomatous disease (1–3), alterations in migration due to leukocyte adhesion deficiency-1 (4), and also lysosomal trafficking in Chediak–Higashi syndrome (5). Neutrophils kill invading pathogens via oxidative mechanisms that involve myeloperoxidase generation of hypochlorous acid and non-oxidative mechanisms that involve cytoplasmic granules that release their content of antimicrobial peptides and enzymes directly into the phagosome (6). In turn, both protease and peroxidase activity is supported by changes in phagosomal pH, which is directed by protons and ions that compensate the electrogenic charge incurred by activation of the nicotinamide adenine dinucleotide phosphate (NADPH) oxidase (7). Moreover, neutrophils can release DNA complexed with cytosolic and granular proteins to the extracellular space, forming neutrophil extracellular traps (NETs) to immobilize and kill pathogens (8).

The azurophilic granules, known also as primary granules, are considered the main microbicidal compartment mobilized upon phagocytosis and contain an array of antimicrobial peptides and serine proteases and also peptidyl-arginine deiminase (PAD) enzymes (9). Neutrophils express PAD isotypes 2 and 4 (10) and, in the presence of calcium, catalyze the irreversible post-translational modification of arginine residues to citrulline, in a process termed citrullination or deimination (11). Several functions of PADs in neutrophils have been described, including a key role for PAD4-induced histone H3 citrullination, essential for chromatin decondensation and NET formation (12). Indeed, citrullination of arginine residues 2, 7, and 17 of histone H3 by PAD4 during NET formation has been described (13). In contrast, although PAD2 can induce histone H3 citrullination, it is considered that it has limited functionality in neutrophils, owing to its low expression (14). Notably, however, localization of PADs within neutrophil cytoplasmic granules suggests an antimicrobial function of PAD enzymes, as previously proposed (15).

Sustained neutrophil activation and aberrant extracellular release of primary granule components can lead to tissue damage in a range of inflammatory conditions. For example, NE is the major damaging protease in the lung causing degradation of structural proteins including elastin of the airways. Moreover, the contribution of PADs to the pathology of disease has been explored in chronic obstructive pulmonary disease (COPD) (9) and rheumatoid arthritis (RA), with 60%–80% of patients with RA presenting with circulating anti-citrullinated protein antibodies (16). Despite these damaging effects outside the cell, however, a clear beneficial role for primary granule components including serine proteases for adequate bacterial killing within the confines of the phagocytic vacuole is well-documented (17). In contrast, an analogous function for PADs within this cell compartment is unexplored. Accordingly, the aim of this study was to investigate the microbial-killing abilities of PADs and to further examine their potential antimicrobial effect within the neutrophil phagosome.

## 2 Materials and methods

### 2.1 Chemicals and reagents

All chemicals and reagents were of the highest purity available and were purchased from Sigma-Aldrich, Ireland, unless indicated otherwise.

### 2.2 Microbial strains and maintenance

*Pseudomonas aeruginosa* (*P. aeruginosa*) (PAO1) (18), *Escherichia coli* (*E. coli*) (ATCC/11775), *Staphylococcus aureus* (*S. aureus*) (ATCC/25923), and *Candida albicans* (*C. albicans*) (MEN) were used in this study. Bacteria and yeast were cultured in Luria-Bertani (LB) broth (Fisher Scientific) and yeast extract peptone dextrose (YPD), respectively at 37°C with shaking at 140 rpm in a Stuart Incubator SI500 Orbital Shaker. LB and YPD plates were prepared by supplementing respective media with 1.5% agar (w/v) (Thermo Scientific) in 90-mm petri dishes (Thermo Scientific). Microorganisms were cultured on agar plates overnight at 37°C in a static Heraeus T6 incubator. For all experiments, log-phase microorganisms were used. Log-phase cells were prepared by inoculating 5 mL of broth with a single colony and incubating for 12–18 h. The initial culture was sub-cultured into fresh broth and incubated for three doubling times to 0.6 ± 0.06 optical density at 600 nm using a SpectraMax M3 microplate reader (Molecular Devices) (19, 20). Microorganisms were stored long term in −80°C in broth with 50% glycerol (v/v). For short-term storage, single colonies streaked on agar plates and stored at 4°C for < 2 weeks.

### 2.3 Neutrophil fractionation

For blood donation, ethical approval was received from the Beaumont Hospital Ethics Board (REC reference #18/52) and informed consent obtained from all healthy participants (n = 11). Human neutrophils were isolated from heparinized (10 U/mL; Sarstedt, Numbrecht, Germany) venous blood as previously described (21) and were resuspended in phosphate-buffered saline (PBS) containing 5 mM glucose (PBSG). Neutrophil preparations were determined 98% pure by flow cytometric analysis, employing a monoclonal antibody to CD16b (22, 23). Neutrophil viability was assessed by trypan blue exclusion assay and found to be >98% pure by use of an Invitrogen CountessTM 3 automated cell counter.

For the preparation of whole-cell lysates, neutrophils (~5 × 10^7^/mL) were gently suspended in Break buffer [10 mM 2,2'-piperazine-1,4-diylbisethanesulfonic acid (PIPES), 3 mM NaCl, 10 mM KCl, and 4 mM MgCl_2_ (pH 7.0)] supplemented with a protease and phosphatase inhibitor cocktail tablet (ThermoFisher) containing aprotinin, bestatin, E-64, leupeptin, sodium fluoride, sodium orthovanadate, sodium pyrophosphate, β-glycerophosphate, and ethylenediaminetetraacetic acid. Neutrophil cell suspension was subjected to N_2_ cavitation under 400 pounds per square inch (psi) for 15 min in a pre-chilled cavitation chamber. Slow release of neutrophils from the chamber into lower atmospheric pressure results in plasma membrane disruption, but internal organelle structures remain intact (24). Neutrophil lysates were collected in a pre-cooled 15-mL falcon tube and centrifuged at 500 x *g* for 5 min at 4°C to obtain the post-nuclear supernatant (PNS).

For isolation of neutrophil granular subtypes and cytosolic fractions, neutrophil lysates were obtained from ~5 × 10^7^ neutrophils. The neutrophil PNS was placed on top of a discontinuous sucrose gradient of 30% and 43% (v/w) in a 13.2-mL, open-top thin-walled Ultra-Clear tube (Beckman Coulter, catalog # 344059). Samples were centrifuged at 285,000 x *g* for 90 min at 4°C in a Beckman Coulter SW40Ti swinging bucker rotor. Cytosolic proteins were harvested from on top of the 30% (w/v) sucrose layer, secondary and tertiary granules were collected from the interface between the 30% and 43% (w/v) sucrose layers, and the primary granules were collected from below the 43% (w/v) layer.

For isolation of neutrophil phagosomes, DynabeadsTM beads pre-coated with recombinant Protein G (Invitrogen) were first prepared following the manufacturer’s protocol. Beads were then opsonized with human serum (60 mg/mL; Invitrogen) overnight at 4°C with gentle rolling and then washed three times with PBS and separated from supernatants using a DynaMag™-2 Magnet (Invitrogen). Neutrophils (1.75 × 10^7^/mL) were incubated with the opsonized magnetic beads (5 mg/mL) for 10 min at 37°C with gentle rotation. Phagocytosis was stopped with ice cold PBS (4× volume). Neutrophils were collected by centrifugation at 500 x *g* for 5 min at 4°C (Eppendorph centrifuge 5417R) and resuspended in Relaxation Buffer [2 mM MgCl_2_, 100 mM KCl, 20 mM PIPES, 1.25 mM EGTA, and 1 mM adenosine tri-phosphate (ATP) (pH 7.2)] supplemented with a cocktail protease and phosphatase inhibitor tablet (Roche). Neutrophils (8.75 × 10^6^/mL) were subjected to N_2_ cavitation using 400 psi for 20 min in a pre-chilled cavitation chamber. A PNS was prepared and exposed to the magnet for 5 min to isolate neutrophil phagosomes that were washed two times with Relaxation Buffer. Phagosomes were denatured in Laemmli Sample Buffer (Bio-Rad) with 5% β-mercaptoethanol (v/v), supplemented with a protease and phosphatase inhibitor tablet, and subjected to Western blot analysis.

### 2.4 Neutrophil activity assays

Neutrophil degranulation assays were performed to investigate extracellular release of PADs. Cells (2 × 10^7^/mL) suspended in PBSG remained unstimulated or stimulated with N-Formylmethionine-leucyl-phenylalanine (fMLP) (1 µM) and/or tumour necrosis factor alpha (TNFα) (10 ng/mL) for 10 min. To stop the reaction, 3 mL of ice cold PBS supplemented with protease and phosphatase inhibitors was added. Neutrophils were pelleted following centrifugation at 500 x *g* for 5 min at 4°C and extracellular supernatants were collected for Western blot analysis.

For extracellular bacterial killing assays, neutrophils from healthy volunteers were suspended in PBSG and stimulated with fMLP (1 µM) and TNFα (10 ng/mL) for 10 min at 37°C and the extracellular supernatants containing degranulated proteins collected by centrifugation as previously described (25). *P. aeruginosa* cells were exposed to the extracellular degranulated material for indicated times (supernatant of 1 × 10^7^ neutrophils per 2 × 10^6^ CFU). In a subset of experiments, PAD activity in degranulated fractions was inhibited by the addition of the PAD2 inhibitor, AFM-30a (Cayman Chemical), or PAD4 inhibitor, GSK484 (Cayman Chemical) (50 µM for 2 min), employing DSMO [0.5% (v/v)] as the solvent control. Bacterial killing was determined by culturing bacteria on LB agar plates and enumerating CFU. In control experiments, recombinant human histone H3 (2 μM) was citrullination *in vitro* in the absence or presence of recombinant (r) PAD2 or rPAD4 (Cayman Chemical) (10 nM) in Tris-NaCl buffer [100 mM Tris, 125 mM NaCl, 1 mM Dithiothreitol (DTT), and Igepal CA-630 0.05% (v/v) (pH 7.4)] supplemented with or without 1 mM CaCl_2_ for 37°C for 1 h (9). In further experiments, the ability of particulate stimuli to cause release of PADs was explored. For this, nutrophils (5 × 10^7^/mL) suspended in PBSG were incubated with non-opsonized *P. aeruginosa* multiplicity of infection (MOI) = 4 or 40) for 10 min. Neutrophils were pelleted following centrifugation at 500 x g for 5 min at 4°C, and extracellular supernatants were collected for Western blot analysis.

Neutrophil phagosomal bacterial killing assays were carried out as previously described (17). In brief, neutrophils (5 × 10^7^/mL) suspended in PBSG were pre-incubated with AMF-30a (50 µM) or GSK484 (50 µM) for 15 min followed by the addition of non-opsonized *P. aeruginosa* (MOI = 4) for 0, 5, 10, 20, or 30 min. For both extracellular and phagosomal bacterial killing, the data were expressed as percentage survival of the original number in PBS.

### 2.5 Electron microscopy

Isolated neutrophils were incubated with immunoglobulin G (IgG)-opsonized bacteria (MOI = 4) for 2 min at 37°C in a stirring chamber. Neutrophils were pelleted at 400 x g for 30 s and transferred to Whatman filter paper to absorb excess water. Samples were frozen in liquid propane using the EMS-002 Rapid Immersion Plunge Freezer (EMS, OA, USA) and transferred into cyro-vials for storage in liquid nitrogen. For cyro-sectioning, heptane was used to fix cell pellets onto sectioning pins that were fastened into the sample holder of an EM UC6 ultratome (Leica, UK). Thin specimens (150 nm) were sectioned at 120°C using a glass knife and collected on 100–150 square or hexagonal mesh nickel grids coated with formvar resin (TAAB-Epon, Agar Scientific UK). Samples were freeze-dried overnight using the K775 freeze dryer (Emitech Ltd., UK), followed by carbon coating to stabilize specimens (25). Prepared sections were examined by TEM (Tecnai T12, FEI Company, Oregon, USA) using an accelerating voltage of 120 kV.

### 2.6 Sodium dodecyl sulfate polyacrylamide gel electrophoresis and Western blotting

Samples were prepared for electrophoresis in Laemmli sample buffer (Bio-Rad) with 5% β-mercaptoethanol (v/v), and boiled at 98°C for 3 min in an Eppendorf Thermomixer compact. Samples were resolved on NuPage 4-12% Bis-Tris gels (Invitrogen), in a mini-gel tank (Invitrogen). Gels were electrophoresized at 114 V with pre-stained protein ladder (3 µL) loaded into at least one well of each gel (Thermo Scientific). Following sodium dodecyl sulfate polyacrylamide gel electrophoresis (SDS-PAGE), gels were stained with Coomassie Blue Stain [45% methanol (v/v), 10% acetic acid (v/v), and 2% Brilliant Blue R (w/v)] for 1 h, with gentle shaking (Stuart gyro-rocker SSL3). Gels were destained using Destain Solution [25% methanol (v/v), 10% acetic acid (v/v), and 65% dH_2_0 (v/v)] and stored long term in 1% acetic acid (v/v) at 4°C.

For Western blot analysis, gels were transferred to 0.2-µm polyvinylidene difluoride (PVDF) membrane (ThermoFisher) in ice cold Transfer Buffer with 20% methanol (v/v), at 250–300 mA for 90 min. PVDF membranes were blocked in Blocking Buffer containing 5% skimmed milk powder (w/v) (Marvel) with or without 1% bovine serum albumin (BSA) (w/v) in Tris-buffered saline [TBS; 15 mM NaCl and 2 mM Tris (pH 7.6)] with 0.1% Tween 20 (v/v) (TBST), for 1 h at room temperature (RT) or overnight at 4°C with gentle shaking. Blots were probed with primary antibodies as described in **Supplementary Table 1** and diluted in 1% skimmed milk powder (w/v) in TBST for 1 h at RT or overnight at 4°C. Blots were washed with TBST for 1 h with gentle shaking, with TBST freshly added every 15 min. The blots were incubated with horse radish peroxidase (HRP)–conjugated secondary antibodies against species-specific IgG (**Supplementary Table 1**), diluted in Blocking Buffer, for 1 h at RT and then washed. Blots were developed using Immobilon Western Chemiluminescent HRP substrate (Merck Millipore). Images were visualized using an Amersham Imager 6000.

### 2.7 The antimicrobial activity of purified recombinant PADs

Bacterial cells were harvested at log phase (0.6 OD_600_) and were centrifuged at 3,500 x *g* for 10 min to pellet cells. The bacterial pellet was washed three times and resuspended in PBS. Survival of *P. aeruginosa* (5 × 10^5^ CFU/mL) was assessed 30 min after treatment with 2.5–40 nM recombinant PAD 2 (rPAD2) or rPAD4, bactericidal permeability increasing protein (rBPI) (Origene), 2.5–1500 nM ciprofloxacin, or 0.05–2.17 nM hexa his tag peptide (Biozol). rBPI was used as a positive control, found in neutrophil primary granules, and previously demonstrated to have potent bactericidal activity against *P. aeruginosa* (5). Bacterial survival was determined by culturing bacteria on LB agar plates and enumeration of CFU. Additional experiments were repeated at pH 5.5, 6.5, 7.5, or 8. In additional experiments, *Staphylococcus aureus* (*S. aureus*) and yeast *Candida albicans* (*C. albicans)* (5 × 10^5^ CFU/mL) was assessed similarly with 20 nM and 200 nM rPAD2 or rPAD4.

### 2.8 Bacterial subcellular fractionation and blot overlay assays

Bacterial lysis was performed as previously described (26). In brief, *P. aeruginosa* cells were harvested at log-phase by centrifugation (5,000 x *g* for 10 min at 4°C), washed three times in PBS and frozen at −20°C. To lyse bacterial cells, the pellet was thawed, resuspended in ice cold lysis buffer [10 mM KCl, 3 mM NaCl, 4 mM MgCl_2_, and 10 mM piperazine-N,N′-bis[2-ethanesulfonic acid] (pH 7.2)] supplemented with protease inhibitors, sonicated six times for 30 s at 80 amps (Sonics Vibra cell™). Following sonication, unlysed cells were removed by centrifugation (5,000 x *g* for 10 min at 4°C). Cell lysates were treated with ribonuclease A (300 μg) and Deoxyribonuclease II (150 μg) for 30 min at RT. Sucrose gradient subcellular fractionation was performed as previously described (27) by centrifugation (186,000 x *g* for 2 h at 4°C) in a Beckman Coulter SW60Ti swing out bucket. Bacterial cell cytosol and cell wall fractions were subjected to SDS-PAGE and transferred to PVDF membrane by Western blotting. Overlay blots were carried out as previously described (26), by incubated with rPAD2 or rPAD4 enzymes (1 µg/mL) in PBS for 2 h at RT. Bound enzymes were detected using anti-his tag protein antibodies (0.1 µg/mL) followed by HRP-conjugated mouse secondary antibodies (**Supplementary Table 1**).

### 2.9 Flow cytometry analysis

*P. aeruginosa* cells (5 × 10^7^ CFU/mL) were fixed in 0.4% (w/v) paraformaldehyde for 15 min at RT. Bacteria were washed two times in PBS following centrifugation at 3,500 x *g* for 10 min, and resuspension in PBS. *P. aeruginosa* (5 × 10^7^ CFU/mL) was incubated with rPAD2 or rPAD4 (0–80 nM) for 30 min, 37°C, and 150 rpm and then washed two times in PBS to remove unbound enzyme. Cells were blocked for 30 min in ice-cold PBS supplemented with 1% BSA (w/v). Subsequently, PAD2 and PAD4 binding was evaluated using anti-PAD2 and anti-PAD4 antibodies for 1 h on ice, respectively, followed by Phycoerythrin (PE)-conjugated mouse anti-rabbit IgG (Santa-Cruz) for 1 h on ice. Control experiments included untreated cells incubated with anti-PAD2 or anti-PAD4 antibodies and/or PE-conjugated mouse anti-rabbit IgG. Samples were analyzed on a Beckman Coulter (CytoFLEX). At least 10,000 events were acquired and the mean fluorescence intensity (MFI) for each experiment was determined using FlowJo^®^ software.

### 2.10 Statistical analysis

Results are expressed as mean ± standard error mean (SEM) of the mean from at least three biological replicates calculated on the average of at least two technical replicates. Statistical analysis of results was performed using GraphPad Prism (v 9.4.1). Comparison between three or more groups was defined using a one-way analysis of a variance (ANOVA) followed by Tukey’s test. Comparison between three or more groups across two parameters was defined using a two-way ANOVA followed by Tukey’s test. A p-value of <0.05 was considered statistically significant.

## 3 Results

### 3.1 PAD2 and PAD4 are localized to neutrophil primary granules and are released extracellularly in response to soluble pro-inflammatory stimuli

**Figure 1:**
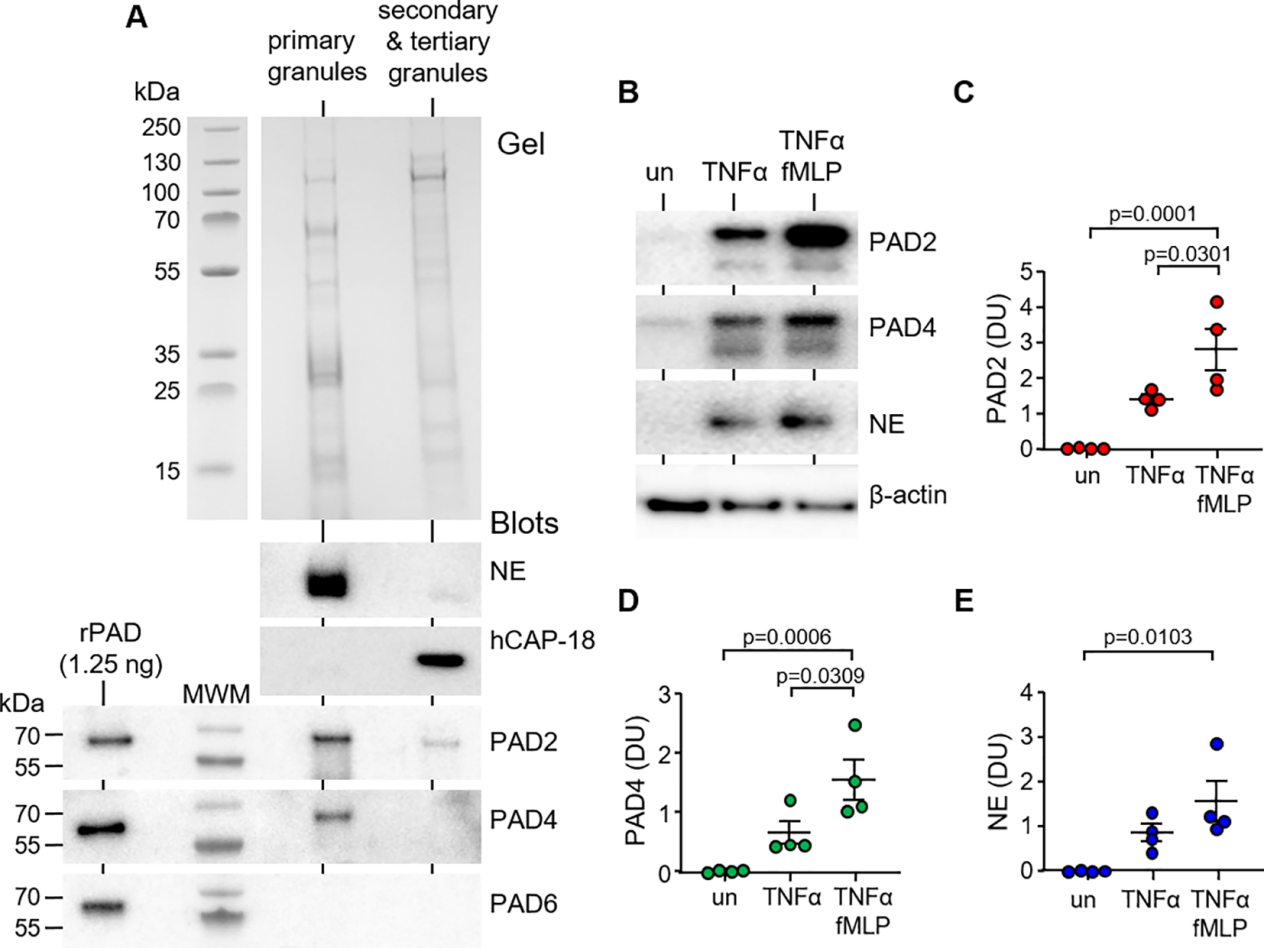
PAD2 and PAD4 are present in primary granules and degranulated extracellularly in response to soluble stimuli. **(A)** Neutrophil granules were isolated by sucrose gradient ultracentrifugation of neutrophil lysates (5 × 10^7^). Coomassie Blue stain of polyacrylamide gel loaded with neutrophil primary granules and secondary and tertiary granules. Western blot detection of PADs in respective fractions, including PAD6 (negative control), primary granule marker neutrophil elastase (NE), and secondary granule marker hCAP-18. Recombinant human proteins of PAD isotypes loaded (1.25 ng) to the left of the molecular weight marker (MWM) as positive controls. Representative images from n = 6 biological repeats. **(B–E)** Neutrophils (1.5 × 10^7^) were left unstimulated (un) or stimulated for 10 min with TNFα or TNFα and fMLP. Extracellular supernatants were assessed by Western blot for PAD2, PAD4, and the primary granule marker, NE. Neutrophil pellets were probed for β-actin (loading control). **(C–E)** Abundance of extracellular PADs was quantified by densitometric analysis of immunobands. Data are expressed as relative densitometry units (DU), with representative Western blots presented (N = 6 biological repeats, one-way ANOVA, followed by Tukey’s *post-hoc* multiple comparison test).

PAD enzymes have previously been identified in cytosol and cytoplasmic granules of neutrophils (28). To confirm the PAD composition of the different granule types, sub-cellular fractionation of neutrophils was performed by sucrose gradient ultracentrifugation. Immunoblotting of cellular fractions identified PAD2 and PAD4 in primary granules of neutrophils, with minor amounts of PAD2 detected in the secondary and tertiary granule fraction (**Figure 1A**). Positive controls included immunoblots for NE and hCAP-18, markers of primary and secondary granules, respectively (29). PAD6 was employed as a negative control, shown previously not to be expressed by neutrophils (30).

The effect of pro-inflammatory stimuli on release of PADs from neutrophils was next investigated. Neutrophils were treated with a combination of TNFα (10 ng) and fMLP (1 µM), which are known potent inducers of the degranulation process (31). In unstimulated neutrophils, and, upon activation, release of PADs from primary granules was detected in the extracellular supernatant by immunoblotting and protein levels evaluated by densitometry. After 15 min of TNFα/fMLP stimulation, a significant increase in extracellular levels of PAD2 was detected compared to that in control unstimulated cells (p = 0.0001) or TNFα treatment (p = 0.0301) (**Figures 1B, C**). A corresponding increase in released PAD4 in the surrounding media was recorded, with a maximum release of PAD4 observed following TNFα/fMLP co-stimulation (p = 0.0006) (**Figures 1B, D**). In corroboration with primary granule degranulation, neutrophils released significant levels of NE in response to TNFa/fMLP treatment (p = 0.103) (**Figures 1B, E**). In further experiments, we explored the extracellular degranulation of PADs in response particulate stimuli. For this, extracellular medium was harvested after 10 min of neutrophil exposure to *P. aeruginosa* at an MOI of either 4 or 40. At an MOI of 4, there was no significant release of PAD2, PAD4, or myeloperoxidase (MPO). An MOI of 40 yielded a significant increase in extracellular levels of PAD2 (p = 0.0452), PAD4 (p = 0.0332), and MPO (p = 0.0437) when compared to that in control unstimulated cells (**Supplementary Figures 1A–D**). Collectively, these results illustrate the presence of PADs in neutrophil primary granules with increased secretion following exposure to pro-inflammatory stimuli. Subsequently, their ability to kill bacteria extracellularly was explored.

### 3.2 The bactericidal action of extracellular PADs is independent of H3 citrullination

**Figure 2:**
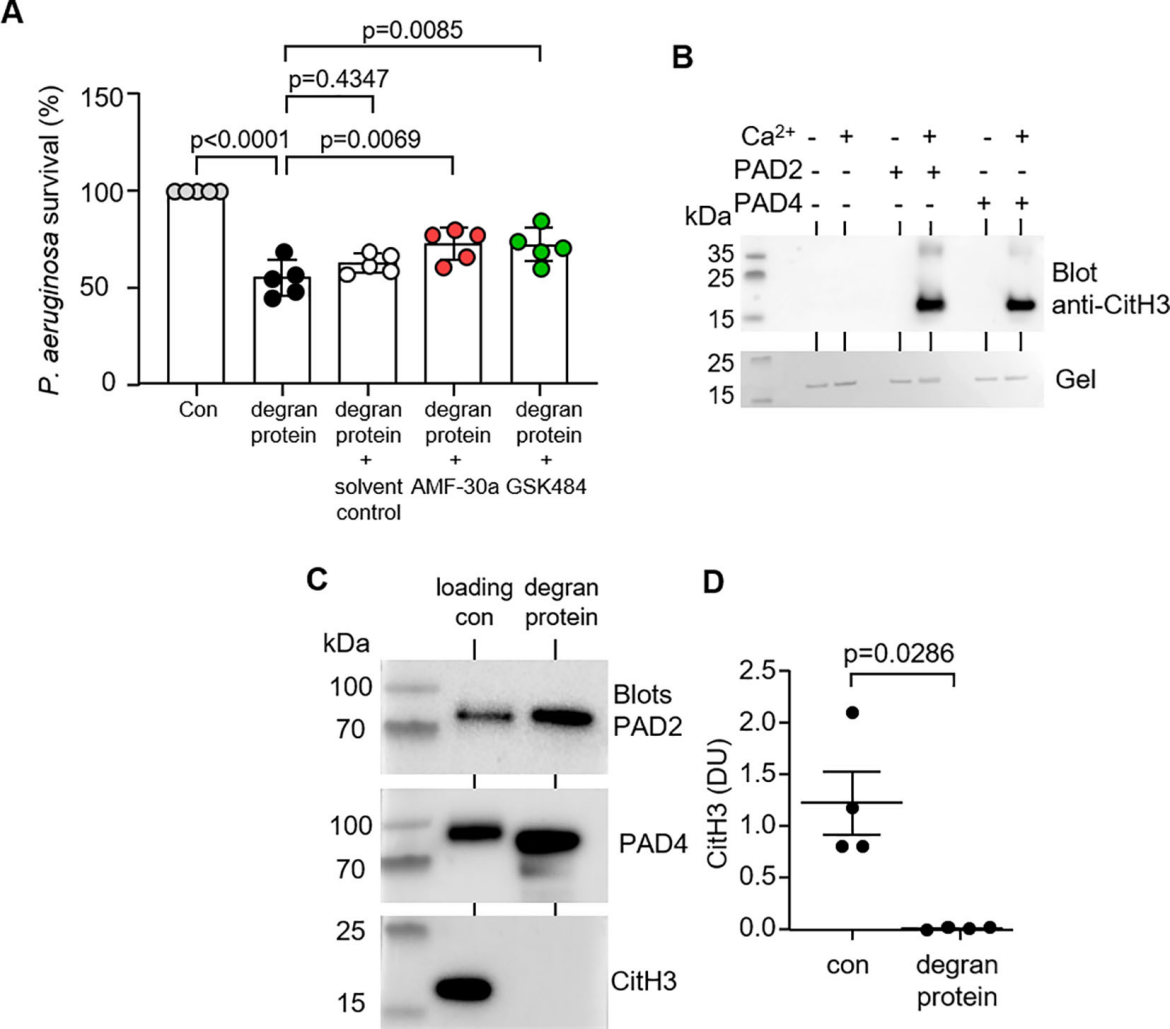
PAD2 and PAD4 released extracellularly impact bacterial killing independent of NET formation. **(A)** Survival of *P. aeruginosa* after 10 min exposure to degranulated proteins obtained from TNFα (10 ng/mL) and fMLP (1 µM) stimulated neutrophils (1.5 × 10^7^, 10 min of stimulation). Extracellular supernatants were treated with DMSO (vehicle control), AMF-30a (PAD2-inhibitor), or GSK484 (PAD4-inhibitor) (50 µM). Data were analyzed by one-way ANOVA followed by multiple comparison Tukey’s *post-hoc* test and presented as mean ± SEM, n = 5 biological replicates. **(B)** Representative Western blot showing citrullination of Histone H3 (CitH3) by PAD2 and PAD4 in the presence of Ca^2+^ (1 mM). Lower panel is a Coomassie Blue–stained gel of reactions, demonstrating equal H3 protein loading. **(C)** Neutrophils (1.5 × 10^7^) were stimulated for 10 min with TNFα and fMLP. Extracellular supernatants of activated cells were probed by Western blot for PAD2, PAD4, and the NET marker CitH3 (right hand lanes). CitH3 was not detected, confirming the absence of NETs in supernatants of activated neutrophils. Loading controls in the left hand lane include rPAD2, rPAD4, and CitH3. **(D)** By densitometric analysis, CitH3 was not detected extracellularly. Data are expressed as relative densitometry units (DU) (N = 4 biological repeats).

Ensuing experiments investigated the antimicrobial impact of neutrophil released PADs. For this analysis, the chosen microbe was the Gram-negative bacterium *P. aeruginosa*, the archetypical bacterial pathogen of the airways, associated with a greater rate of decline of lung function (32). To assess whether neutrophil degranulated PADs were capable of killing *P. aeruginosa*, bacteria were exposed to degranulated protein material in the presence or absence of PAD2 or PAD4 small-molecule inhibitors, AFM-30a and GSK484, respectively. It has previously been shown that GSK484 demonstrates a high affinity for PAD4, binding adjacent to the active site forming a β-hairpin (33), and AFM-30a orientates into a hydrophiobic binding region of PAD2 within the C-terminal domain (34). Relative to the untreated bacteria, *P. aeruginosa* (2 × 10^6^ CFU/mL) exposed to degranulated proteins of 1 × 10^7^/mL neutrophils for 10 min demonstrated just 54.7 ± 9.2% survival (p < 0.0001) (**Figure 2A**). In turn, addition of either AFM-30a or GSK484 significantly increased *P. aeruginosa* survival compared to uninhibited degranulated protein samples. Inhibition of PAD2 or PAD4 increased bacterial survival to 72.3 ± 8.2% (p = 0.0069) and 71.8 ± 8.7% (p = 0.0085), respectively. Controls for this experiment included the addition of dimethyl sulfoxide (DMSO) (solvent control), with no significant impact on *P. aeruginosa* survival relative to untreated degranulation protein solution (p = 0.4347).

PADs are key enzymes regulating NET’s skeleton protein histone H3 to citrulline histone (CitH3). This process is Ca^2+^ dependent and is inhibited by the addition of either AFM-30a or GSK484 (**Figure 2B**). To exclude the possibility that PAD-induced CitH3 and NETs were responsible for *P. aeruginosa* killing within the experimental design, extracellular supernatants of TNFα/fMLP-stimulated neutrophils were probed for CitH3 (**Figure 2C**). The observation that significantly elevated levels of PAD2 and PAD4 were detected extracellularly, contrasts the results demonstrating an absence of H3cit (**Figures 2C, D**). Controls for this experiment included enzymatic citrullination of H3 by rPAD2 and rPAD4 *in vitro*. Overall, these results indicate that PAD2 or PAD4 is involved in the antimicrobial processes of neutrophils, and the significant decrease in *P. aeruginosa* survival was independent of the PAD-CitH3 pathway.

### 3.3 Intra-phagosomal degranulated PADs participate in bacterial killing

**Figure 3:**
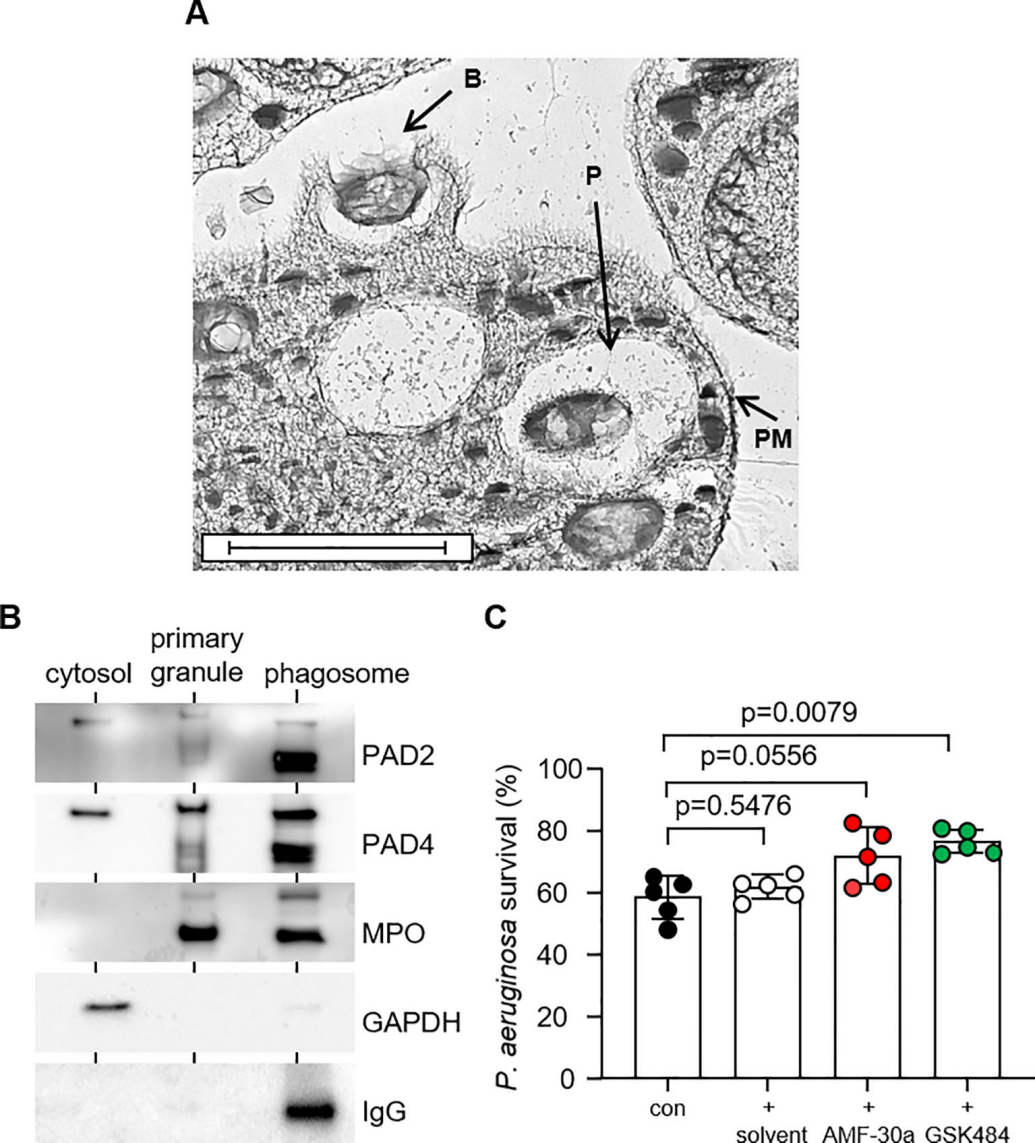
PAD2 and PAD4 are degranulated into the bacterial phagosome and participate in bacterial killing. **(A)** Representative micrograph showing neutrophil forming pseudopodia at the time of engulfment and bacteria inside phagocytic vacuoles following 3 min of incubation [Labels indicate bacteria **(B)**, phagosomes (P), and plasma membrane (PM); scale bar, 2 μm]. **(B)** Western blot analysis of neutrophil cytosol, primary granules and phagosome fractions for PADs, including primary granule and phagosome marker MPO, and cytosolic marker GAPDH. Representative images of n = 3 biological replicates. **(C)** *P. aeruginosa* survival following 30 min of neutrophil (5 × 10^7^/mL) phagosomal killing (MOI = 4; 1 neutrophil to 4 bacteria) (con). Neutrophils were pre-treated with PAD inhibitors AMF-30a (50 µM) or GSK484 (50 µM) for 15 min prior to killing assays. DMSO [0.5% (v/v)] was used as the solvent control. Data were analyzed by a one-way ANOVA followed by multiple comparison Tukey’s *post-hoc* test and presented as mean ± SEM, n = 5 biological replicates.

On encountering bacteria, neutrophils form pseudopodia, which surround and engulf the microbe within phagocytic vacuoles, which fuse with intracellular granules (35). The moment of engulfment and formation of phagocytic vacuoles was captured by EM (**Figure 3A**), and this cell compartment is the focus of this study. Accordingly, the aim of this experiment was to investigate PAD enzyme degranulation in to phagocytic vacuoles. Following engulfment of IgG-opsonized Dynabeads for 10 min, purified vacuoles were analyzed for the presence of PAD2 and PAD4, employing vacuolar myeloperoxidase (MPO) as a marker of primary granule intra-phagosomal-degranulation. An immunoband in response to anti human-IgG antibody confirmed phagocytosis of IgG-opsonized beads and purity of phagocytic vacuolar fractions. Glyceraldehyde 3-phosphate dehydrogenase (GAPDH) was used as a cytosolic protein marker (36), and confirmed PAD2 and PAD4 in this subcellular fraction. Finally, as per the MPO profile, both PAD2 and PAD4 were detected in primary granules and vacuolar fractions, confirming degranulation and release of PADs into this subcellular compartment (**Figure 3B**).

Neutrophil intra-phagosomal killing of bacteria is a rapid process (17), with greater than 60% of microbes killed within minutes (37). This was confirmed in neutrophil-mediated *P. aeruginosa* killing assays of the current study, with 41.48 ± 6.85% killing recorded 30 min after phagocytosis (**Figure 3C**). To understand the impact of PAD2 and PAD4 on this vacuolar bactericidal process, neutrophils were pre-incubated with AMF-30a or GSK484, respectively. Exposure of neutrophils to GSK484 prior to carrying out the killing assays resulted in a significant 18.03 ± 3.83% increase in bacterial survival when compared with cells not exposed to the PAD4 inhibitor (p = 0.0079). Moreover, neutrophils that were exposed to the PAD2 inhibitor, AMF-30a, also demonstrated a decreased ability to kill *P. aeruginosa* successfully, with a 13.43 ± 9.09% increase in bacterial survival recorded, although this did not reach significance. Controls for this latter experiment included the addition of the solvent control DMSO, with no significant impact on intra-phagosomal *P. aeruginosa* survival (p = 0.5476). Moreover, as a previous study demonstrated that AFM-30a and GSK199 impacted *E. coli* antibiotic sensitivity (38), in further control experiments, we assessed the direct effect of the inhibitors AFM-30a and GSK484 (50 µM) against *P. aeruginosa* (2 × 10^6^ CFU) viability. Whereas AFM-30a minimally reduced *P. aeruginosa* survival (p = 0.0032), neither DMSO nor GSK484 impacted bacterial survival (**Supplementary Figure 2**). Overall, these results indicate that PADs positively impact on the vacuolar ability of neutrophils to kill bacteria.

### 3.4 PADs bind directly to the outer surface of *P. aeruginosa*

**Figure 4:**
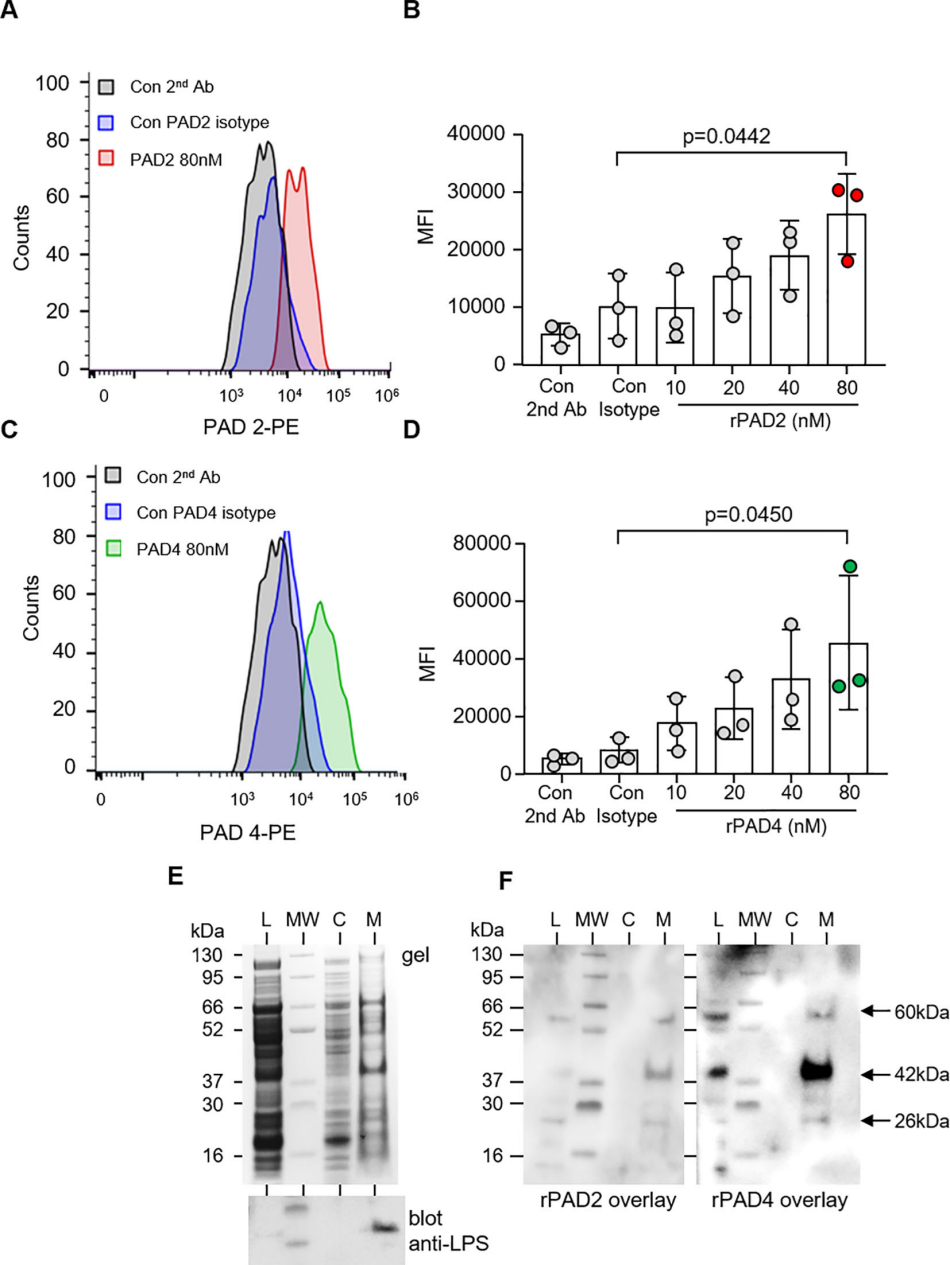
PAD2 and PAD4 directly bind *P. aeruginosa.* Flow cytometry analysis of PAD2 **(A, B)** and PAD4 **(C, D)** (10–80 nM) membrane interaction with *P. aeruginosa* (5 × 10^7^ CFU) after 30 min of incubation at 37°C. rPAD2 and rPAD4 binding was evaluated using anti-PAD2 and anti-PAD4 rabbit polyclonal antibodies, respectfully, followed by use of anti-rabbit PE-conjugated antibody. Data were analyzed by one-way ANOVA followed by multiple comparison Tukey’s *post-hoc* test and presented as mean ± SEM, n = 3 biological replicates. **(E)** Subcellular fractions of *P. aeruginosa* [bacterial whole-cell lysate (L), cytosol (C), and membrane proteins (M)] were electrophoresed on a polyacrylamide gel and stained with Coomassie Blue (top panel). A Western blot for *P. aeruginosa* LPS confirmed purity of membrane proteins (lower panel). **(F)** The subcellular distribution of the bacterial adhesion to which PADs bound was determined after transfer to PVDF membrane and incubation with rPAD2 or rPAD4. Arrows indicate protein bands of ∼60, 42, and 26 kilodaltons in the outer membrane fractions corresponding to molecules with which rPAD2 and rPAD4 interacted.

To confirm that PADs interact with *P. aeruginosa*, the binding of PAD enzymes to the surface of bacteria was assessed by flow cytometry. Specificity for binding events was confirmed as no significant difference in MFI was observed when bacteria were exposed to secondary antibody only, or PAD2 (p = 0.8972) and PAD4 isotype control antibodies (p = 0.997). Relative to the respective isotype control, 80 nM PAD2 (p = 0.0262) (**Figures 4A, B**) and PAD4 (p = 0.0467) showed significant binding to *P. aeruginosa* (**Figures 4C, D**).

To further characterize *P. aeruginosa* adhesins that interact with PADs, whole bacterial lysate was subjected to subcellular fractionation to yield a cytosol and a membrane fraction. Purity of *P. aeruginosa* outer membranes separated by sucrose gradient ultracentrifugation was verified by the distribution of lipopolysaccharide (LPS) (**Figure 4E**, lower panel). Subsequently, fractions of *P. aeruginosa* were electrophoresed on polyacrylamide gels, transferred to PVDF membrane, and incubated with rPADs. rPAD2 and rPAD4 both interacted with three molecules with molecular mass of approximately 26, 42, and 60 kilodaltons. The three binding molecules were detected in the outer membrane fractions of the organism (**Figure 4F**) and were not detected in cytosolic fractions or in control blots employing primary and secondary antibodies only (result not shown), indicating that this reaction was a specific membrane interaction with PADs.

### 3.5 Recombinant human PAD enzymes possess antibacterial activity against *P. aeruginosa in vitro*, an effect increased at neutral-alkaline pH

To further characterize the cytotoxicity of PADs, we explored their effect against additional bacteria and also fungi, including Gram-positive bacterium *S. aureus* and yeast *C. albicans.* In *in vitro* experiments, *S. aureus* and *C. albicans* cells were exposed to increasing concentrations of rPADs for 30 min (0 nM, 20 nM, and 200 nM). Relative to the untreated control, at 200 nM PAD enzyme, *S. aureus* subjected to either rPAD2 (p = 0.8887) or rPAD4 (p = 0.6837) showed no reduction in survival (**Supplementary Figure 3A**). Likewise, neither PAD2 (p = 0.3798) nor PAD4 (p > 0.999) impacted *C. albicans* survival (**Supplementary Figure 3B**).

**Figure 5:**
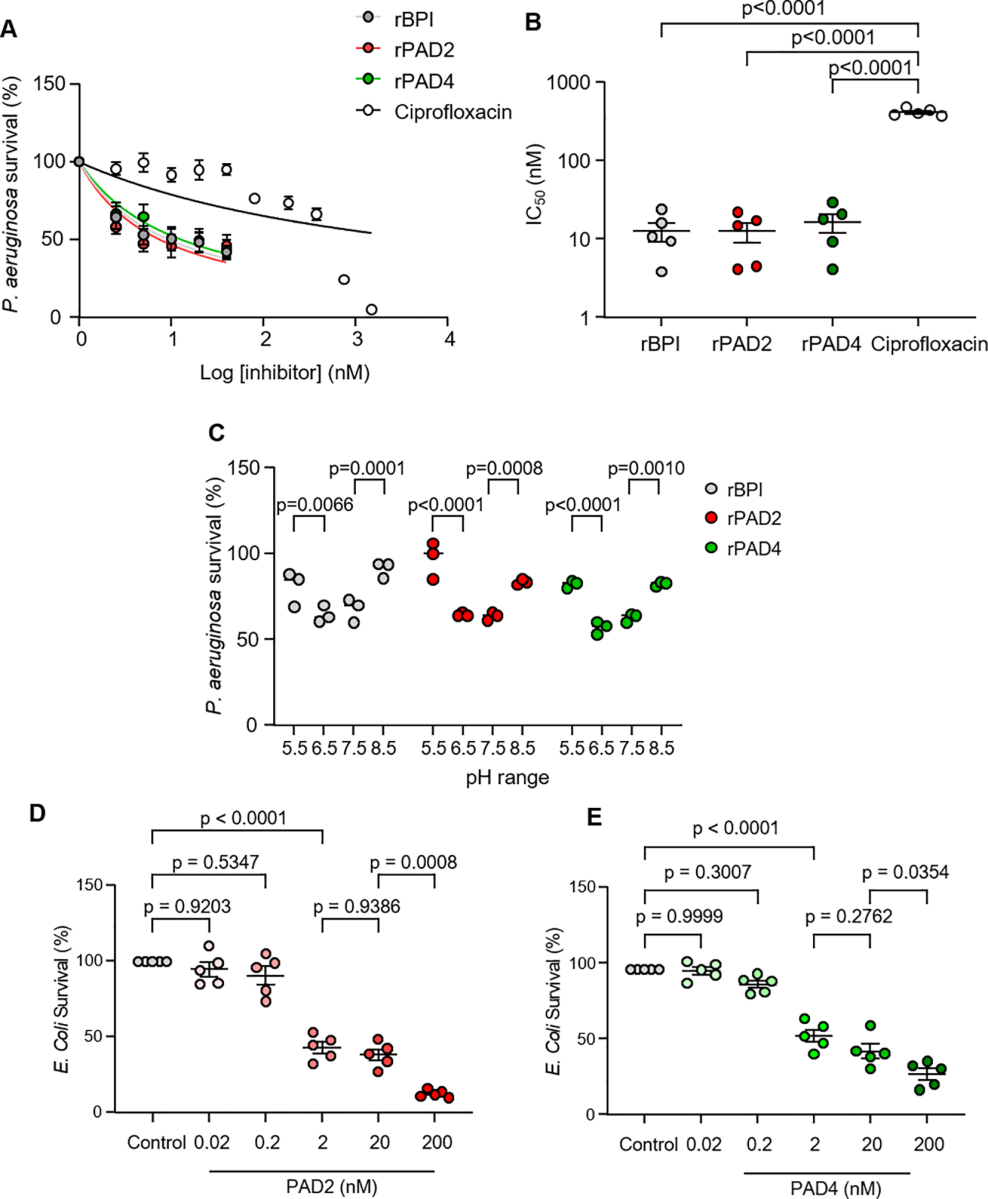
Recombinant human PAD2 and PAD demonstrate dose-dependent killing of *P. aeruginosa* and *E coli*. **(A)** Survival of *P. aeruginosa* (5 × 10^5^ CFU/mL) 30 min after incubation with rPAD2, rPAD4, rBPI (2.5–40 nM), or ciprofloxacin (2.5–1500 nM) in PBS (pH 7.4). Survival (%) calculated relative to untreated *P. aeruginosa*. Data expressed as a non-linear regression and presented as mean ± SEM, n = 5 biological replicates repeats. **(B)** Compounds were tested for activity against *P. aeruginosa* and MIC_50_ values calculated. **(C)** Survival of *P. aeruginosa* (5 × 10^5^ CFU/mL) 30 min after incubation with rPAD2, rPAD4, or rBPI (20 nM) in PBS (pH 5.5, 6.5, 7.5, or 8.5). Survival (%) calculated relative to *P. aeruginosa* CFU/mL in 0 nM enzyme at respective pH levels. **(D, E)** Survival of *E Coli* (5 × 10^5^ CFU/mL) 60 min after incubation with rPAD2 **(D)** or rPAD4 **(E)** in PBS (pH 7.4) (n = 5 biological replicates). Data were analyzed by one-way or two-way ANOVA, followed by multiple comparison Tukey’s *post-hoc* test and presented as mean ± SEM.

Ensuing experiments studied the antibacterial activity of PADS against *P. aeruginosa*, in comparison to ciprofloxacin. Ciprofloxacin is a known antimicrobial protein in neutrophils and therapeutic antibiotic that demonstrates potent activity against *P. aeruginosa* (39). The minimum inhibitory concentration (50% inhibition) of PADs and ciprofloxacin used for antibiotic comparisons are shown in **Figure 5**. Similar to the antimicrobial effect of rBPI, bacteria were extremely sensitive to rPADs, and, after just 30 min of incubation, BPI, PAD2, and PAD4 displayed MIC50 values of 12.6 ± 7.5 nM, 12.4 ± 7.9 nM, and 16.1 ± 9.8 nM, respectively (**Figures 5A, B**). For the same experimental time point of 30 min, ciprofloxacin displayed an MIC50 value of 396.3 ± 30.9 nM against *P. aeruginosa*. PAD2, PAD4, and BPI showed no significant difference in MIC50 values, but all demonstrated a significantly lower MIC50 value than ciprofloxacin (p < 0.0001) (**Figure 5B**). As rPADs employed in killing assays were histidine-tagged (his-tag) proteins, control experiments explored the antimicrobial impact of equal-molar his-tag peptides, with no effect on *P. aeruginosa* survival observed (**Supplementary Figure 4**).

Next, we explored the impact of pH on the anti-*pseudomonal* activity of rPAD2 and rPAD4, as the pH of the neutrophil phagocytic vacuole ranges from 5.5 to 8.5 during the bacterial killing process (17). In control experiments, the anti-bacterial effect of rPADs was compared to that of BPI (10 µg/mL), previously shown effective against Gram-negative bacteria (40). The bactericidal activity of rPAD2, rPAD4, and rBPI were assessed at pH 5.5, 6.5, 7.5, and 8.5 (**Figure 5C**). Results demonstrate that rBPI caused a significant reduction in *P. aeruginosa* survival at pH 7.5 and 6.5 with ~40% killing observed at pH 6.5 (p < 0.0001). In turn, rPAD2 (20 nM) possessed no bactericidal activity at pH 5.5 but demonstrated optimal activity at pH 6.5 (p < 0.0001) and 7.5 (p < 0.0001). Similarly, rPAD4 showed optimal activity at pH 6.5 (p < 0.0001) and 7.5 (p < 0.0001). Collectively, these results highlight the similar anti-*pseduomonal* properties of both rPAD2 and rPAD4, akin to that of rBPI, supporting an important role for PADs in the neutrophil phagosomal killing processes. Furthermore, the antibacterial activity of PADs against an additional Gram-negative bacteria was assessed. PAD2 and PAD4 demonstrated dose-dependent killing of *E. coli* (**Figures 5D, E**). At 2 nM, PAD2 and PAD4 reduced *E. coli* survival to 43.2 ± 3.7% and 56.1 ± 4.1% (p < 0.0001), respectively.

## 4 Discussion

This study demonstrates that PAD enzymes, isotypes 2 and 4, are localized predominantly in neutrophil primary granules and are degranulated extracellularly or into the phagosome following soluble or particulate stimuli, respectively. Application of PAD2 and PAD4 specific small-molecule inhibitors reduced neutrophil extracellular and phagosomal killing of *P. aeruginosa*. Moreover, human rPAD2 and rPAD4 enzymes bind to and possess bactericidal activity against *P. aeruginosa*. These results indicate that PAD2 and PAD4 function as antimicrobial proteins within neutrophils.

The first step in neutrophil phagocytosis involves targeting of opsonized bacteria by several receptors on the outer plasma membrane. These include Fcγ receptors, FcγRIIA(CD32), and also FcγRIIIB (CD16b), which recognize IgG antibodies bound to the bacteria (41, 42) and also complement receptors such as CR1 (CD35) and CR3 (CD18/CD11b), which bind the complement factors C3b and C4b (43). After receptor ligand binding and formation of encircling pseudopodia, targeted bacteria are taken up within a phagocytic vacuole and primary granules fuse with and release their content of enzymes into the phagosome within seconds (44). This process is vitally dependent on the small guanine triphosphate hydrolases Rac2, as evident by human neutrophils dominant negative for Rac2 D57N, which fail to release primary granule components (45). In contrast, increased primary granule release by neutrophils of patients with cystic fibrosis most likely involves increased Rac2 activation (46). The presence of PADs in neutrophil granules has been investigated previously, with consensus on their presence in granular compartments (47) yet disparity on the granule type. In this regard, PAD4 was identified in secondary and tertiary granules (28), yet, in an additional study, both PAD2 and PAD4 were localized predominantly to the primary granules of neutrophils (9), a result in agreement with the current study. Consequently, upon exposure to soluble stimuli including TNFα that primes cells for primary granule degranulation in response to fMLP (48), both PAD2 and PAD4 were released extracellularly. Additionally, in confirmation of degranulation of PADs within phagocytic vacuoles, the phagosomal content of purified neutrophil phagosomes proved positive for both PAD2 and PAD4, indicating that PADs may play a role in microbial killing. Primary granules are considered as the true microbicidal compartment mobilized upon phagocytosis as their content of enzymes and antimicrobial peptides are directly toxic to microbes. For example, NE found in primary granules of neutrophils (49) can cleave bacterial proteins (50) and also plays a critical role in activation of matrix metalloproteases that are produced in an inactive precursor form (51) including matrix metalloproteinase-9 (MMP-9) (52). An interesting point for discussion is whether the phagocytosis of opsonized beads versus bacteria in the current study could lead to differences in phagosome PAD composition. Indeed, as phagocytosis of IgG-opsonized particles (53) and degranulation of primary granules is a rapid process (54) and as neutrophil responses are further amplified by cell priming (55), opsonins and cytosolic-free calcium (56), there are multiple factors that may impact phagosomal PAD levels, necessitating further exploration.

Following identification of the degranulation of PADs, the current study shows that small-molecule inhibitors of PAD2 and PAD4, AFM-30a and GSK484, respectively, reduced killing of *P. aeruginosa*. Of importance, no known adverse effects or off-targets for AFM-30a or GSK484 have been noted (57), which is in contrast to the pan-PAD inhibitor BB-Cl-amidine, which was cytotoxic to T cells, B cells, monocytes, and natural killer cells. Furthermore, rPADs showed antimicrobial activity against Gram-negative *P. aeruginosa* and *E. coli* but no activity against *S. aureus* or *C. albicans* in the current study, possibly indicating that PADs possess a Gram-negative bacteria–type–specific response. It must be noted, however, that PAD4 knockout mice demonstrate no effect on the phagosomal killing of *Shigella flexneri* (12). Moreover, the involvement of PADs in the antimicrobial capacity of neutrophils may either be through association with cytosolic components of the NADPH-oxidase as recently shown (14) or alternatively though direct PAD interaction as we have demonstrated here. Neutrophil phagosomal killing of ingested pathogens lasts up to 20–30 min (58), and, within this timeframe, we demonstrate that recombinant human PAD2 and PAD4 enzymes bind *P. aeruginosa in vitro* and reduce bacterial survival.

To further characterize PAD-induced bacterial killing, we compared PADs to the well-known anti-*pseudomonal* capability of BPI, which by binding LPS in the outer membrane of *P. aeruginosa*, can destabilize the inner membrane, resulting in cell lysis (59). Assays were performed over a pH range as it has previously been shown that, following phagocytosis, a transient increase in pH to 7.8–8.0 occurs within the first 2 min, which is followed by a slow fall to 6.0–6.5 (60, 61). Acidification of phagosomes plays a key role in bacterial killing via MPO, whereby ingested bacteria are subjected to MPO catalyzed halogenation, a process dependent upon the availability of chloride ions and low pH. Whereas MPO-mediated chlorination is maximal at pH 5–6, in contrast, cathepsin G and NE show maximal activity in an alkaline environment of pH 7–9 and pH 8–10, respectively (62). In the current study, PAD2 demonstrated bactericidal activity from pH 6.5–8.5, and PAD4 had a broader range at 5.5–8.5, but, for both enzymes, bactericidal activity was maximal at pH 6.5–7.5. The limitations of this study are the unexplored elements of the antimicrobial mechanism(s) of PADs, which may include direct citrullination of bacterial proteins to which we have shown PADs bind, thereby changing the permeability of the bacterial cell surface. Alternatively, PADs may exhibit activity similar to that of neutrophil lactoferrin, as several antibacterial peptides within the *N*-terminal region of lactoferrin have been shown to be highly active and membrane interactive (63). Further studies are underway to address these questions and also to access the activity of PADs against clinically relevant isolates of *P. aeruginosa*.

Although primary granule components play an essential role in microbial killing, they are also implicated in the pathology of human disease. For example, MPO released extracellularly is capable of enhancing oxidative damage to epithelial cells (64, 65). Moreover, NE released from degranulating or necrotic neutrophils is the major damaging protease in the lung causing degradation of structural extracellular matrix proteins and proteoglycans (66, 67). With regard to PADs, PAD2- and PAD4-induced citrullinated elastin is most susceptible to proteolytic degradation, with implications for patients with COPD (9). These later studies indicate promise for the future development of PAD-based therapeutics for RA and for preserving lung function in patients with COPD, with the caveat of targeting extracellular PAD activity while preserving PAD antimicrobial activity within neutrophil phagosomes.

In conclusion, PAD2 and PAD4 are localized in neutrophil granules, and are degranulated into the phagosome where they participate in the killing of *P. aeruginosa*. Recombinant human PAD enzymes also exhibit robust antimicrobial activity toward *P. aeruginosa*. Together, these data support additional investigations into the therapeutic application of PADs to the treatment of *Pseudomonas* and other Gram-negative bacterial infections. Such studies are particularly important in conditions such as cystic fibrosis, where *P. aeruginosa* is the most prevalent Gram-negative bacterial pathogen (68).

## Data Availability

The raw data supporting the conclusions of this article will be made available by the authors, without undue reservation.
