# Supplementary material for: The vacuolar anti-Pseudomonal activity of neutrophil primary granule peptidyl-arginine deiminase enzymes
Source: Front Immunol. 2024 Oct 18;15:1452393. doi: 10.3389/fimmu.2024.1452393 (PMC11527647; doi:10.3389/fimmu.2024.1452393)
Supplement: Supplementary file 1 [file DataSheet1.docx]

Supplementary Material

Supplementary Data


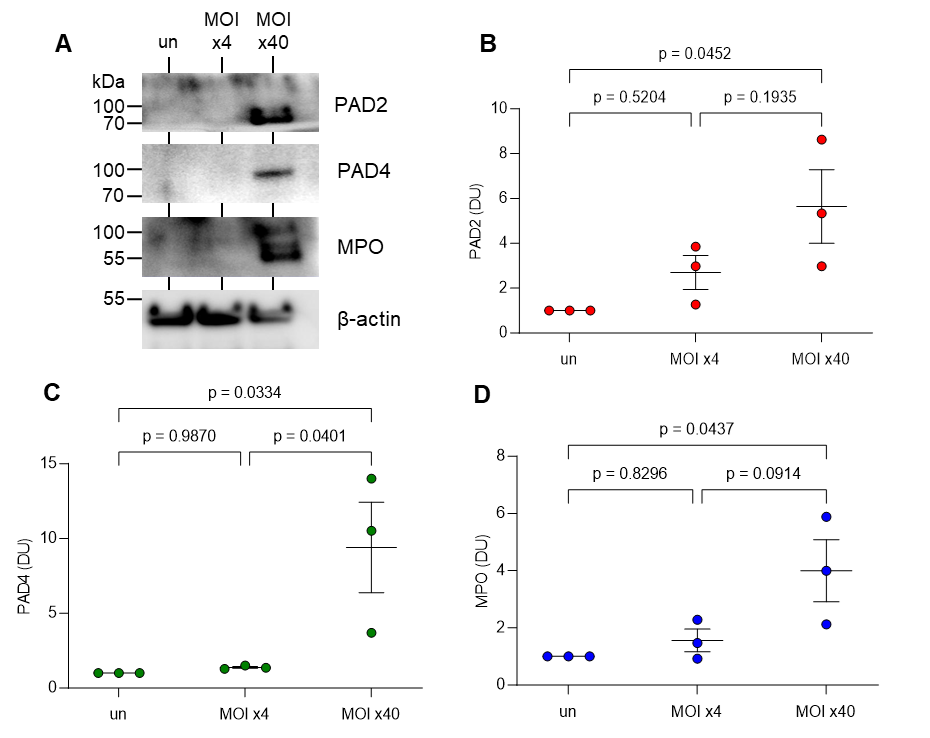


**Supplementary Figure 1. PAD2 and PAD4 are degranulated from neutrophils following *P. aeruginosa* stimulation ex vivo.** Neutrophils (5x10^7^) were left unstimulated (un) or stimulated for 10 min with *P. aeruginosa* (MOI of 4 or MOI of 40). Extracellular supernatants were assessed by western blot for PAD2, PAD4, and the primary granule marker, MPO. Neutrophil pellets were probed for β-actin (loading control). Abundance of extracellular PADs was quantified by densitometric analysis of immunobands. Data are expressed as relative densitometry units (DU), with representative Western blots presented (N=3 biological repeats, one-way ANOVA, followed by Tukeys’ post-hoc multiple comparison test).

**Supplementary Figure 2.** **AFM-30a shows bactericidal activity against *P. aeruginosa***

Survival of *P. aeruginosa* (2x10^6^ CFU) post-10 min exposure to DMSO (0.5% (v/v)) (solvent control), AMF-30a or GSK484 (50 µM) in PBS, pH 7.4. Survival (%) calculated by CFU/mL relative to untreated control. Data was analysed by one-way ANOVA followed by multiple comparison Tukey’s post hoc test and presented as mean ± SEM, n=3 biological replicates.


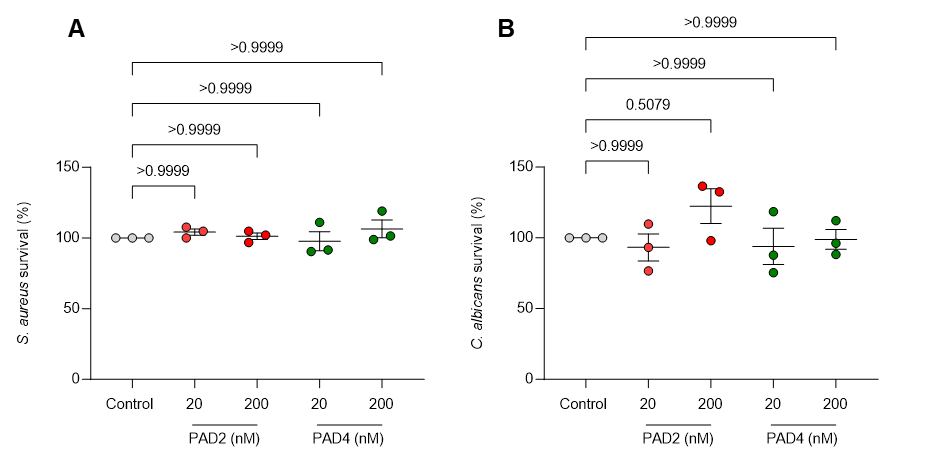


**Supplementary Figure 3.** **PADs show no antimicrobial activity against *S. aureus* and *C. albicans*.**

**A)** Survival of *S. aureus* (5x10^5^ CFU/mL) and **B)** *C. albicans* (5x10^5^ CFU/mL) 30 min post-incubation with rPAD2 or rPAD4 (20 and 200 nM) in PBS, pH 7.4. Survival (%) calculated by CFU/mL relative to untreated control. Data was analysed by one-way ANOVA, followed by multiple comparison Tukey’s post hoc test and presented as mean ± SEM, n=3 biological replicates.

**Supplementary Figure 4. Hexa his-tag peptides have no effect on *P. aeruginosa* survival**

Survival of *P. aeruginosa* (5x10^5^ CFU/mL) 30 min post-incubation with Hexa his tag peptide (0.05 – 2.17 nM) in PBS, pH 7.4. Survival (%) calculated relative to *P. aeruginosa* CFU/mL untreated control (no His tag peptide). Data was analysed by one-way ANOVA, followed by multiple comparison Tukey’s post hoc test and presented as mean ± SEM, n=5 biological replicates.

**Supplementary Methodology**

**Supplementary Table 1: Antibodies used in this study for immunoblotting**

| **Primary antibodies** | **Species** | **Supplier** | **Working concentration (**μg/mL) | **Catalogue #** |
| --- | --- | --- | --- | --- |
| GAPDH | Mouse | Santa Cruz Biotechnology | 0.200 | sc-47724 |
| hCAP-18 | Rabbit | Novus Biologicals | 1.000 | NBP1-76864 |
| MPO | Rabbit | Novus Biologicals | 1.000 | NB600-923 |
| NE | Rabbit | Abcam | 1.000 | ab68672 |
| PAD2 | Rabbit | Cayman Chemical | 1.000 | 33887 |
| PAD4 | Rabbit | Cayman Chemical | 1.000 | 33914 |
| PAD6 | Mouse | Cayman Chemical | 1.000 | 25965 |
| LPS | Mouse | ProteoGenix | 1.060 | SAA0575 |
| His-tag | Mouse | Calbiochem | 0.100 | OB05 |
| Histone H3 (Citrullinated R2 + R8 + R17) | Rabbit | CaymanChem | 0.400 | 17855 |
| β-actin | Rabbit | Cell Signalling Technology | 0.037 | 8457 |
| IgG heavy chain HRP-conjugated | Goat | Santa Cruz Biotechnology | 0.400 | Sc-2453 |
| **Secondary antibodies** | **Species** | **Supplier** | **Working dilution** | **Catalogue #** |
| Mouse IgG HRP-conjugated | Horse | Cell Signalling Technology | 0.092 | 7076S |
| Rabbit IgG HRP-conjugated | Goat | Cell Signalling Technology | 0.039 | 7074S |
